# Supplementary material for: Memory phototransistors based on exponential-association photoelectric conversion law
Source: Nat Commun. 2019 Mar 20;10:1294. doi: 10.1038/s41467-019-09206-w (PMC6426936; doi:10.1038/s41467-019-09206-w)
Supplement: Supplementary file 1 — Supplementary Information [file 41467_2019_9206_MOESM1_ESM.pdf]

# Supplementary Information

# Memory Phototransistors Based on Exponential-Association Photoelectric Conversion Law

Shao *et al.*

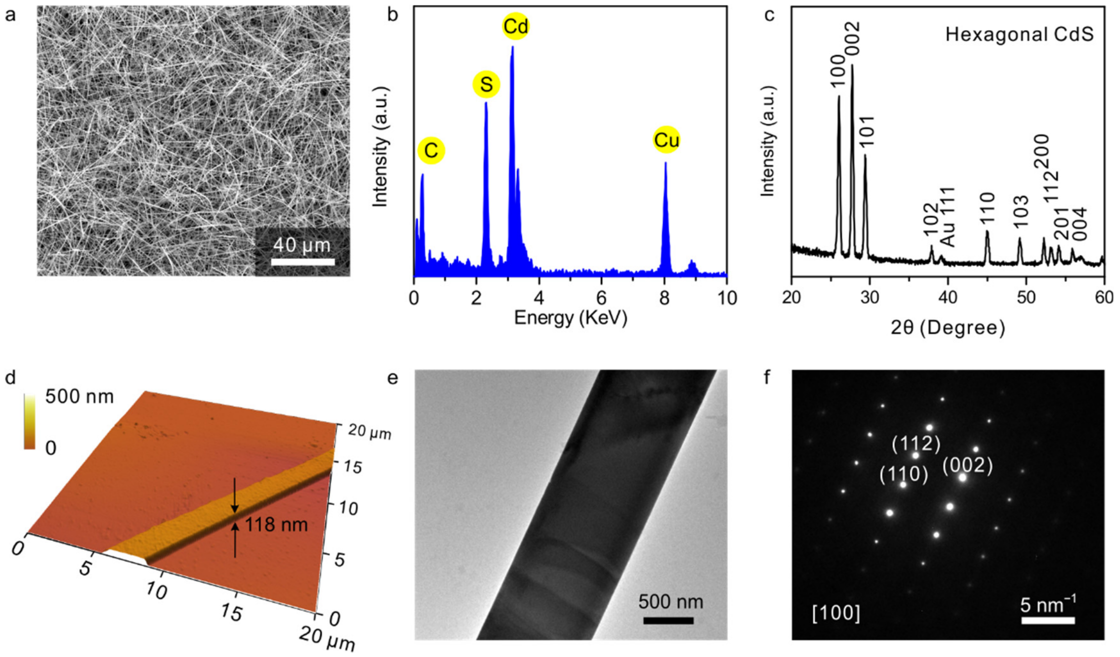


**Supplementary Figure 1** Characterizations of CdS NRs synthesized by physical vapor deposition. (a) SEM image, (b) EDS spectrum, (c) XRD pattern of the CdS NRs. (d) AFM three-dimensional (3D) morphology image, (e) TEM image, and (f) SAED pattern of an individual CdS NR.

Supplementary Figure 1a shows the SEM image of the as-prepared CdS NRs, revealing that a host of CdS NRs with a width of 0.4–5 μm and a length of up to hundreds of micrometers were synthesized. From the EDS spectrum and XRD pattern in Supplementary Figures 1b and 1c, respectively, it can be deduced that the NRs are hexagonal CdS. AFM image measurement indicates a thickness in the range of 100–150 nm for the CdS NRs (Supplementary Figure 1d). Supplementary Figures 1e and 1f show the TEM image and corresponding SAED pattern, respectively, of a single CdS NR. The CdS NR possesses single-crystalline wurtzite structure with a growth orientation of [001]. The top and bottom surfaces of the CdS NR are deduced to be (100) facets.

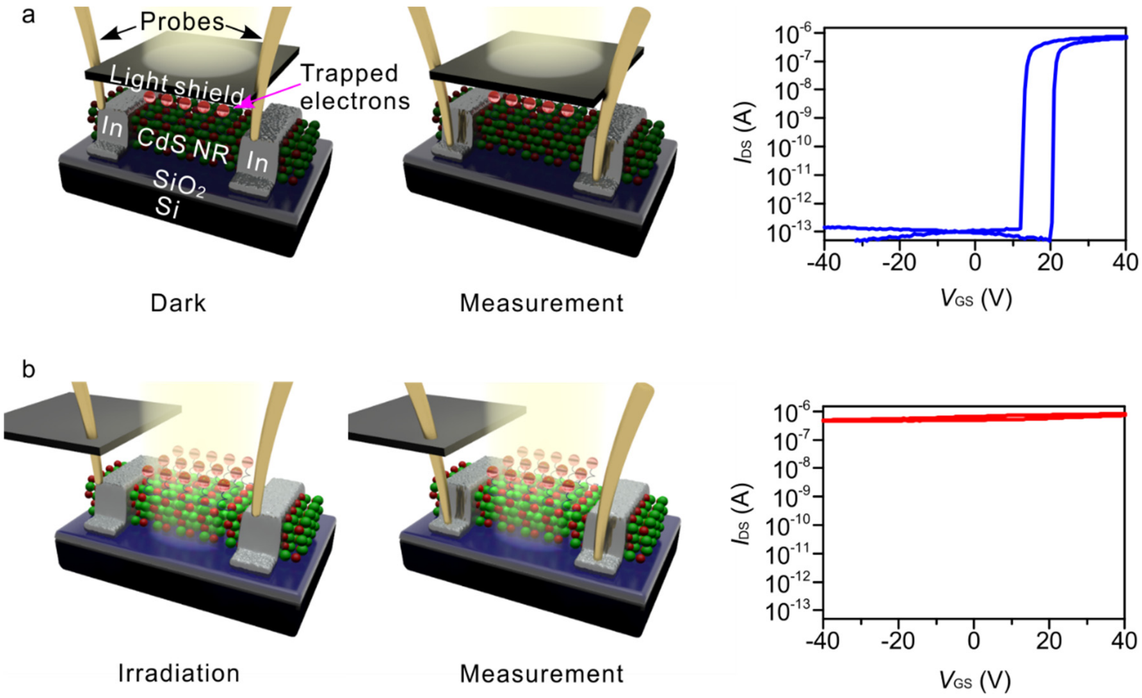


**Supplementary Figure 2** Schematic illustrations and electrical transfer characteristics of CdS NR-based phototransistor device measured (a) in the dark and (b) under light irradiation (100 μW cm$^{-2}$) at a fixed drain voltage of 0.6 V.

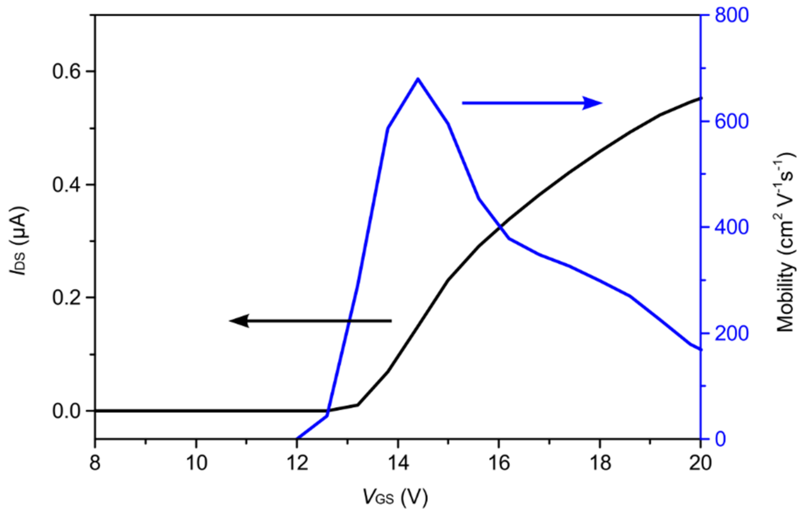


**Supplementary Figure 3** Electrical transfer characteristic in a linear plot (black line) and the extracted field-effect mobility as a function of $V_{GS}$ (blue line) of the CdS NR transistor. The device is measured in the dark at a fixed drain voltage of 0.6 V.

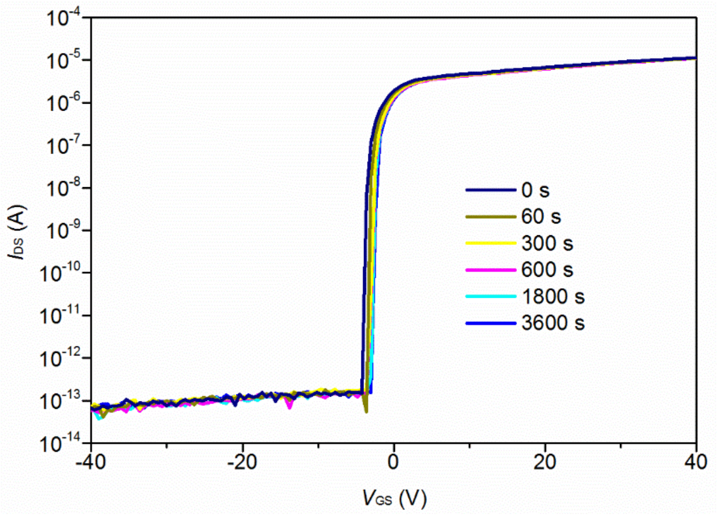


**Supplementary Figure 4** Electrical transfer characteristics in positive sweep measured after 0–3600 s of delay after light irradiation.

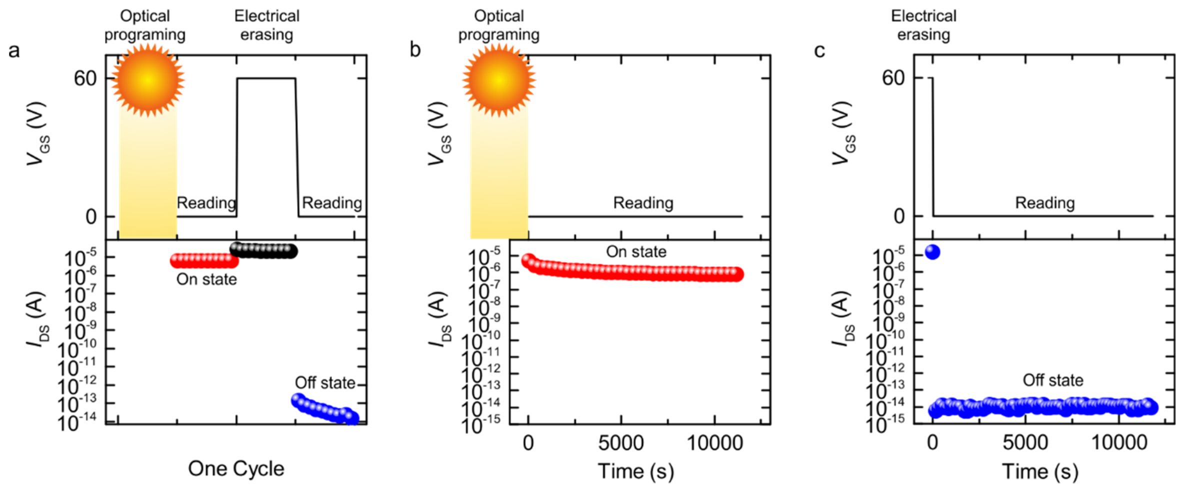


**Supplementary Figure 5** (a) One memory access cycle consisting of optical programing, reading, electrical erasing, and reading. After irradiating the device with light of 190 nW cm$^{-2}$ for 10 s, the $I_{DS}$ of the phototransistor device was recorded in sequence at a reading voltage of 0 V, an erasing voltage of +60 V, and a reading voltage of 0 V, respectively. (b,c) Retention characterization of the ON and OFF states of the phototransistor device. The ON and OFF currents were recorded at a reading voltage of 0 V after applying the optical programming and electrical erasing inputs.

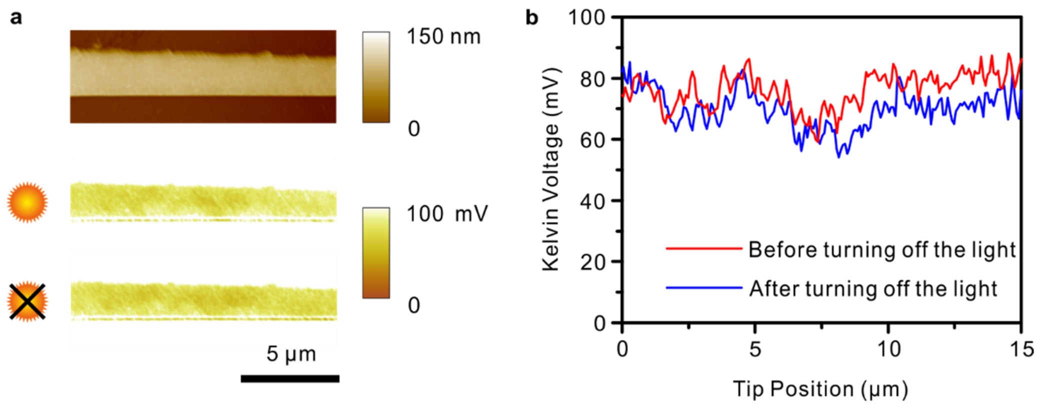


**Supplementary Figure 6** (a) Top: 2D topography image of the CdS NR. Middle: Kelvin potential image of CdS NR measured before turning off the light. Bottom: Kelvin potential image of the CdS NR measured after turning off the light. (b) Line profiles of Kelvin voltage extracted from the potential images measured before (red) and after turning off the light (blue), respectively.

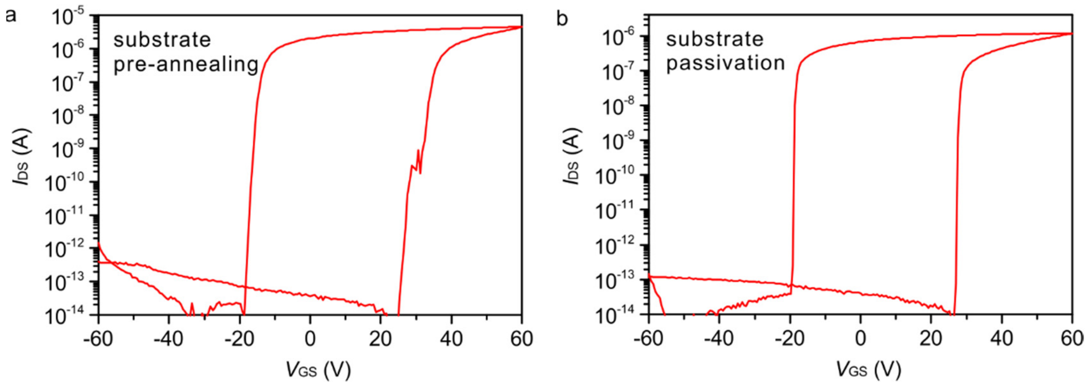


**Supplementary Figure 7** Electrical transfer characteristics of the CdS NR phototransistor fabricated on the pre-treated substrates. The substrate was (a) pre-annealed in $H_2$/Ar at 1100 ºC for 2h or (b) passivated with 10 nm thick $Al_2O_3$.

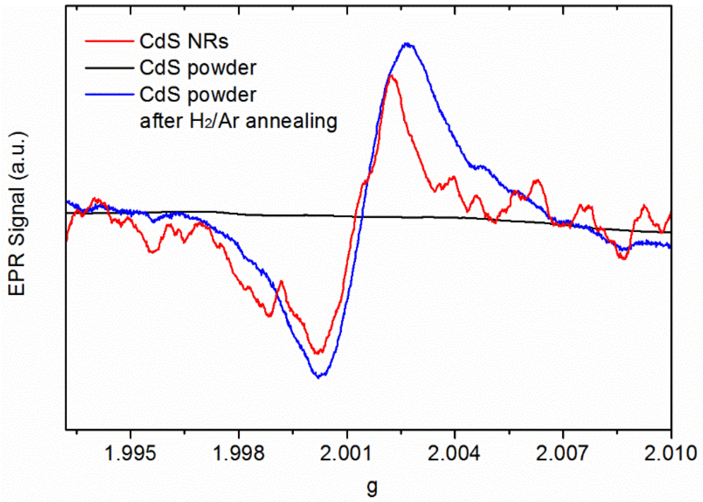


**Supplementary Figure 8** EPR spectrum of CdS NRs. For comparison, the spectra of CdS powder before and after $H_2$/Ar annealing at 800 °C for 1 h were also measured.

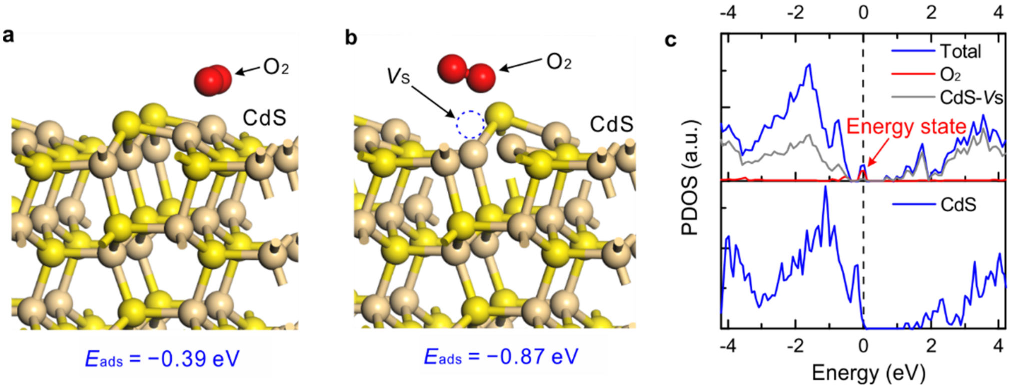


**Supplementary Figure 9** (a,b) Energetically favorable configurations of one oxygen molecule-adsorbed CdS surface without and with sulfur vacancy, respectively. The adsorption energy $E_{\mathrm{ads}}$ is defined by the following equation: $E_{\mathrm{ads}} = E_{\mathrm{O_2-CdS}} - E_{\mathrm{CdS}} - E_{\mathrm{O_2}}$, where $E_{\mathrm{O_2-CdS}}$, $E_{\mathrm{CdS}}$, and $E_{\mathrm{O_2}}$ represent the energy of the full adsorption system, the CdS crystal, and the adsorbed $O_2$ molecule, respectively. (c) Total density of states (DOS, blue lines) spectra of CdS before and after the oxygen adsorption. In the top, the gray and red lines represent the projected density of states (PDOS) of CdS and $O_2$ in the adsorption system, respectively. The arrow indicates the surface energy state introduced from the adsorbed oxygen molecule.

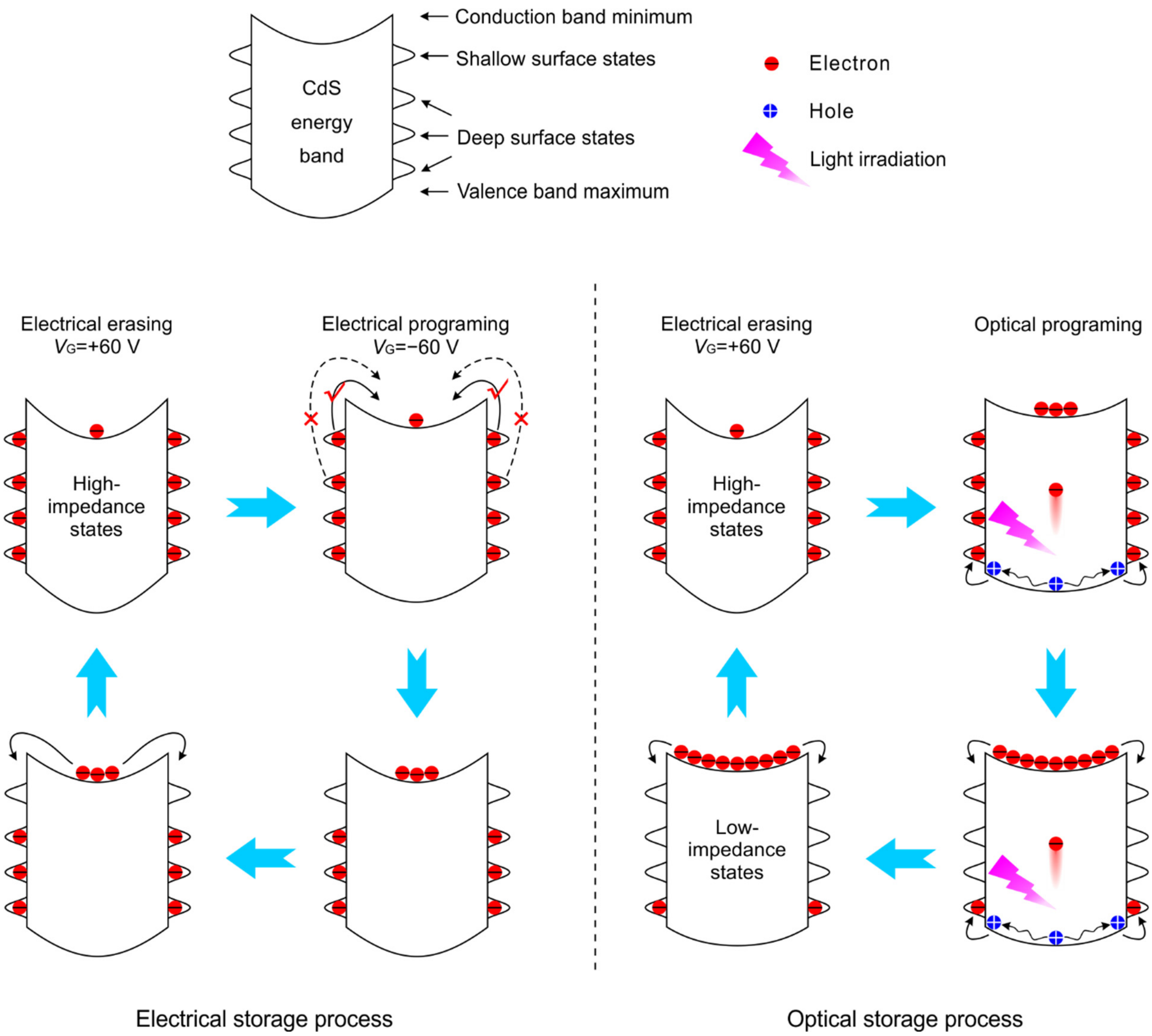


**Supplementary Figure 10** Energy band diagrams of CdS NR during electrical storage process and optical storage process.

Group II-VI semiconductor nanostructures are very sensitive to the surface adsorbates (e.g. oxygen, halogens). So far, the effect of oxygen adsorption on the electrical properties of semiconductors has been extensively studied.[1-3] When II-VI semiconductors adsorb oxygen molecules onto their surface, adsorbed oxygen molecules will fill the oxygen or sulfur vacancies at the surface and form a series of shallow and deep energy states in forbidden gap at the surface.[4-7] Electrons can be trapped by the surface states of CdS NR, resulting in an upward band bending from bulk to surface as well as a high-

impedance state of CdS NR. When a programing voltage of −60 V was applied, electrons in shallow energy states on the surface would jump over the energy barrier into the CdS NR under electric field. However, due to higher energy barrier, electrons in deep energy states on the surface could not be injected into CdS NR. Therefore, under the electrical input excitation, only a small number of shallow energy states could serve as storage sites in CdS NR, resulting in a small storage window of hysteresis curve of CdS NR. On the other hand, under light irradiation, electron-hole pairs were generated in CdS NR. The photogenerated electrons would jump into the conduction band of CdS NR, while the photogenerated holes would be attracted by electrons in the surface states and then recombined with the surface electrons. Under continuous irradiation, the number of electrons in CdS conduction band would increase and gradually tend to be saturated. Due to thermal motion of electrons, a small amount of electrons would jump over cocked energy band at CdS surface and then be trapped by the surface states. At meantime, the photogenerated holes were still generated and recombined with the surface electrons. When the rate of charge recombination was equal to the rate of the electron transition back to surface states, a dynamic equilibrium would be achieved, forming a low-impedance state of CdS NR. When an erasing voltage of +60 V was applied, the electrons in conduction band would jump over cocked energy band under electric field and inject into surface states, returning the device to the high-impedance state. Therefore, under the optical input excitation, all surface states could serve as storage sites in CdS NR, resulting in a larger storage window of hysteresis curve of CdS NR.

To improve the detection capability for light signals, more storage sites that are not interfered by electrical signals are required for accumulating the charges introduced by optical signals. In this case, the devices should possess weak electrical storage capability and strong optical storage capability. In our work, because of large energy barrier between deep energy states and conduction band at the

surface of CdS NR, electrons trapped in deep energy states cannot jump to conduction band under the excitation of programing voltage. All of the deep energy states on the surface could be used to capture the photogenerated charges, resulting in weak electrical storage capability and strong optical storage capability. Nevertheless, in the traditional floating-gate optoelectronic memories, the energy alignment between the storage media and semiconductor conduction band can be easily modulated by the gate voltage.[8, 9] The floating storage media are available for charges induced by both electrical and optical input signals, thus resulting in a strong mutual interference between the electrical and optical input signals. The lack of independent storage media, which are only used for charge storage introduced by the optical signals, will limit the photodetection performance of the traditional floating-gate optoelectronic memories.

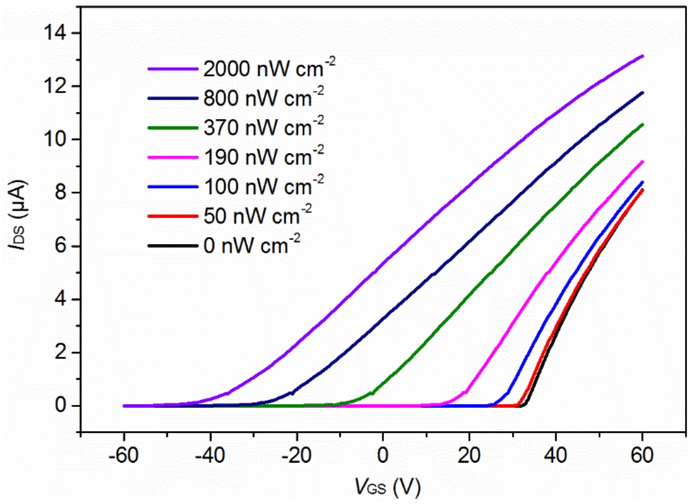


**Supplementary Figure 11** Electrical transfer characteristics of a CdS NR MPT in linear plots. The characteristics were measured in the dark at a fixed drain voltage of 0.6 V, after the MPT was irradiated with light of 0–2000 nW cm$^{-2}$ for 10 s.

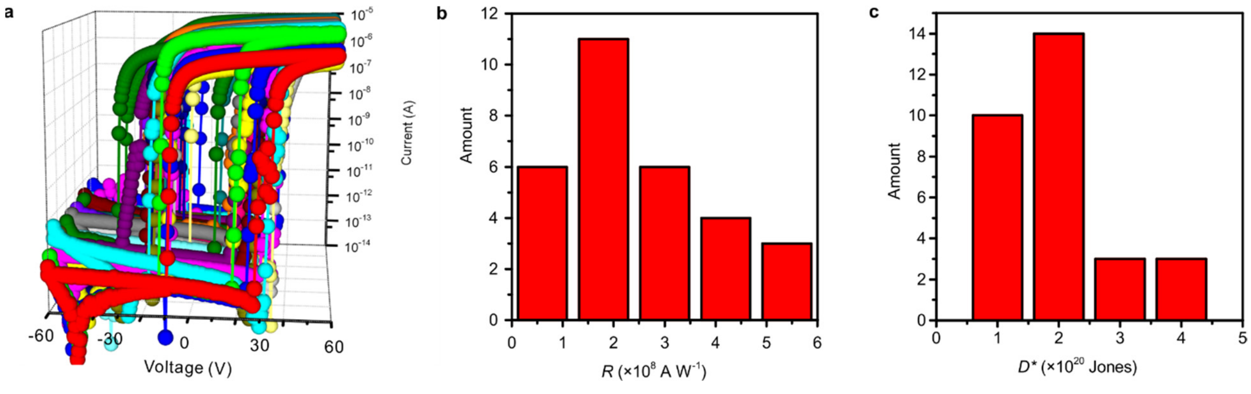


**Supplementary Figure 12** (a) Electrical transfer characteristic curves of 30 CdS NR phototransistors measured in the dark at a fixed drain voltage of 0.6 V, afterwards they were irradiated with light (190 nW cm$^{-2}$) for 10 s. (b,c) Statistical histograms of responsivities and detectivities for these 30 CdS NR phototransistors. The data are extracted from (a).

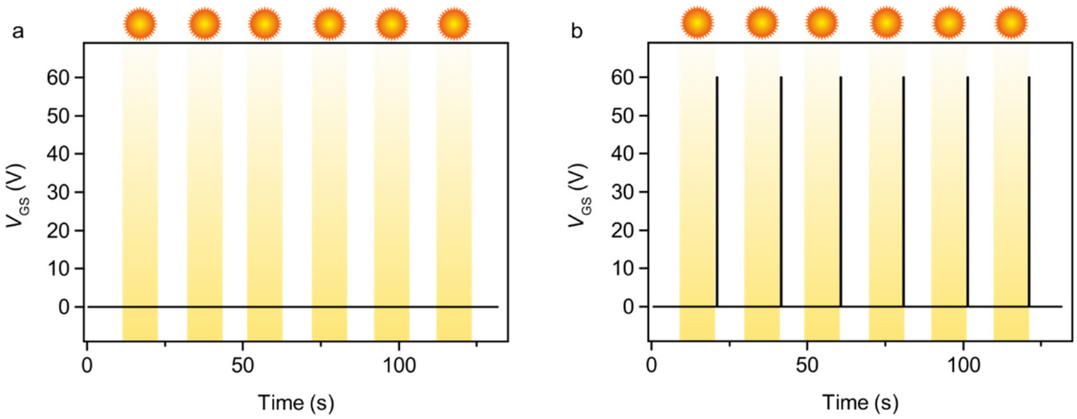


**Supplementary Figure 13** (a) The gate voltage was fixed at 0 V. (b) The periodic erase pulses of +60 V were applied.

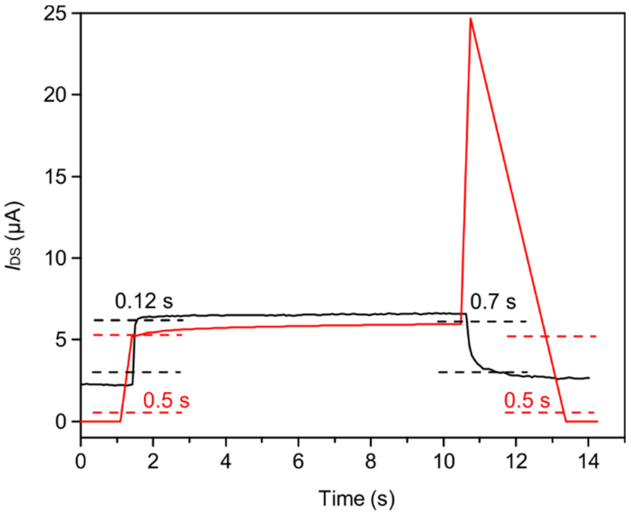


**Supplementary Figure 14** Enlarged rise and fall edges of the temporal response in Figure 3g.

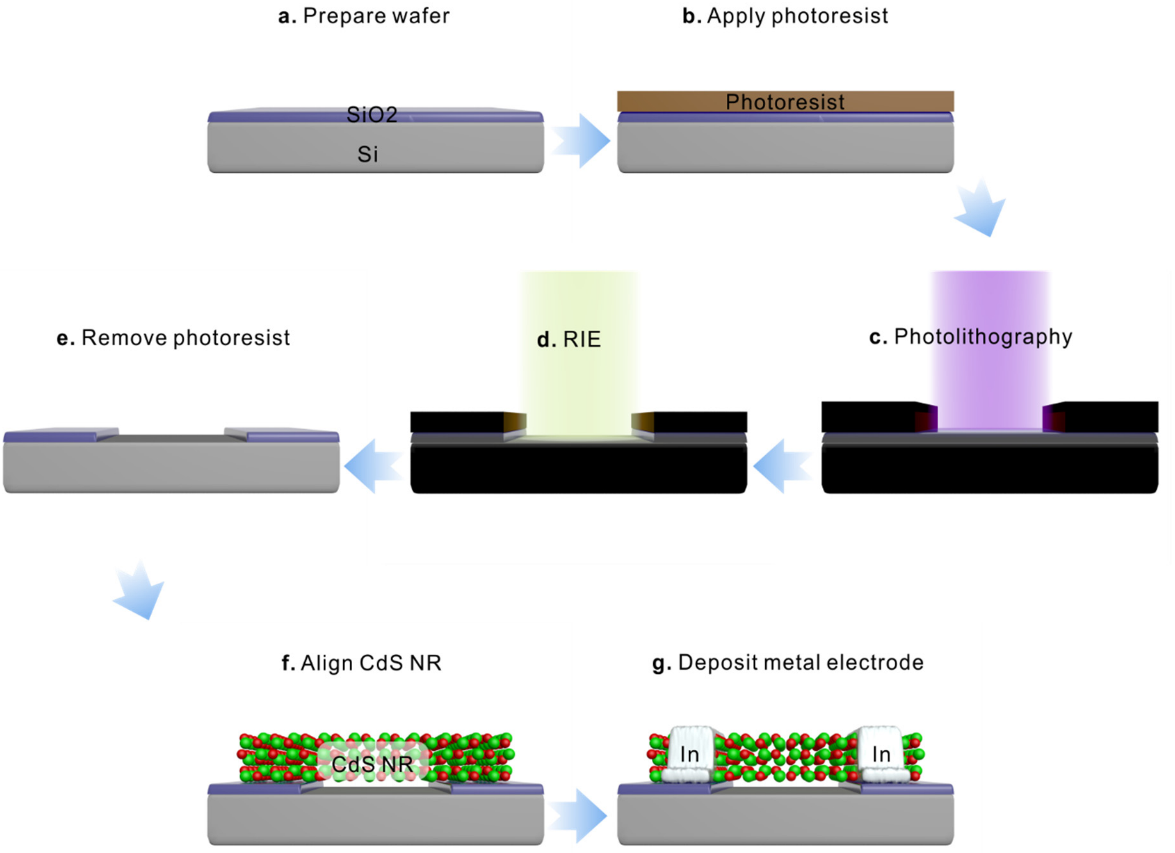


**Supplementary Figure 15** Flow chart showing the fabrication process for a suspended CdS NR MPT. A positive photoresist (Allresist AR-P 5350) was spin coated on the surface of a cleaned $SiO_2$ (300 nm)/$n^+$-Si substrate. After the photolithography and subsequent development process, a photoresist window with 8 μm width was exposed. The $SiO_2$ layer within the photoresist window was then etched by reactive ion etching (RIE). After that, the remaining photoresist was fully removed by immersing the sample in acetone for 5 mins, forming a $SiO_2$ channel on the substrate surface. A CdS NR was aligned vertically across the etched channel. Finally, symmetric indium (200 nm) source and drain electrodes with 20 μm spacing were defined on suspended CdS NR by photolithography, metal evaporation, and lift-off process.

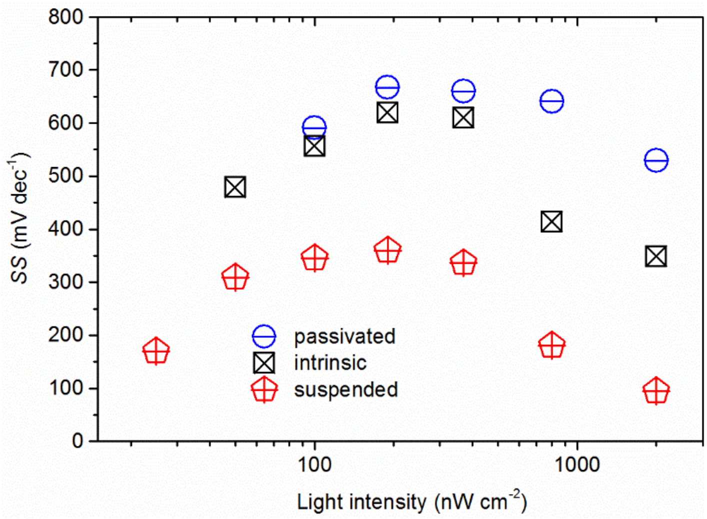


**Supplementary Figure 16** Subthreshold swings of a passivated, normal, and suspended CdS NR MPT at different light intensities.

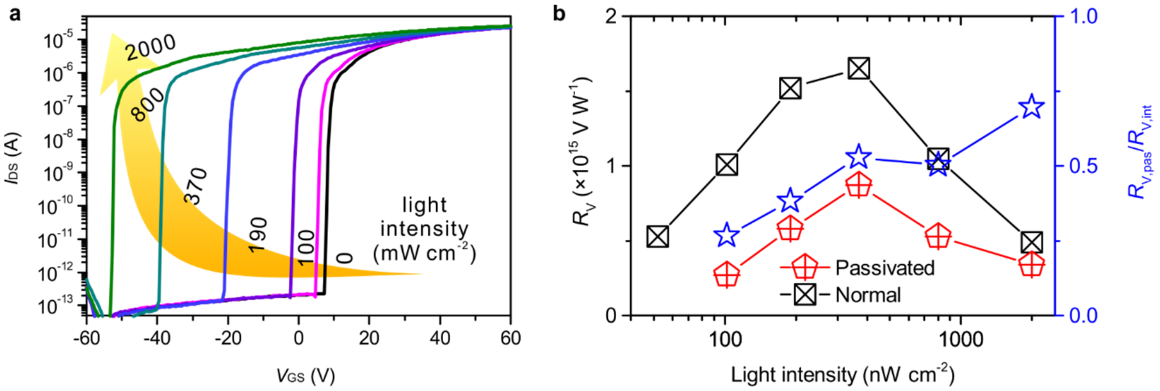


**Supplementary Figure 17** (a) Electrical transfer characteristics of the CdS NR MPT after $Al_2O_3$ passivation. The MPT was measured in the dark after 10 s of light irradiation with different light intensities of 0–2000 nW cm$^{-2}$. (d) Voltage responsivities of normal and passivated CdS NR MPTs, as well as their ratio at different light intensities.

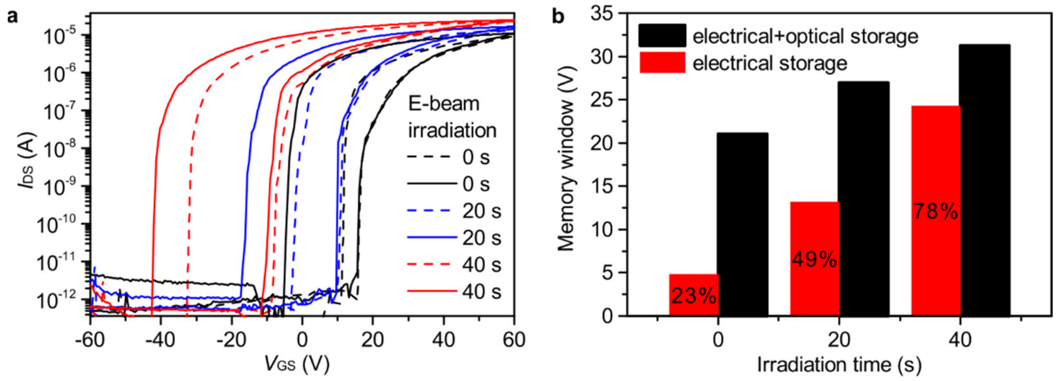


**Supplementary Figure 18** (a) Electrical transfer characteristics of CdS MPT after 0, 20, and 40 s of electron-beam irradiation. The CdS MPT was uniformly irradiated with an electron beam in a SEM with $4\times10^6$ electrons $\mu m^{-2}$ (30 kV accelerating voltage, 223 pA current, 9.9 nm spot size for scanning). The device was measured in the dark with (solid line) and without (dash line) the light pre-irradiation (100 nW $cm^{-2}$) for 10 s. (b) Statistical histograms of memory windows extracted from (a).

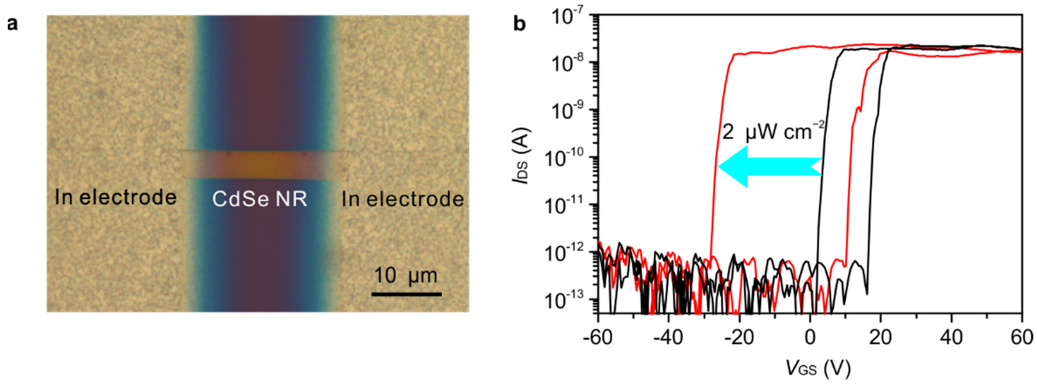


**Supplementary Figure 19** (a) SEM image of the CdSe NR-based MPT device. (b) Electrical transfer characteristics of the CdSe NR MPT measured in the dark at a fixed drain voltage of 0.6 V, with (red curve) and without (dark curve) 10 s light pre-irradiation (2 μW $cm^{-2}$).

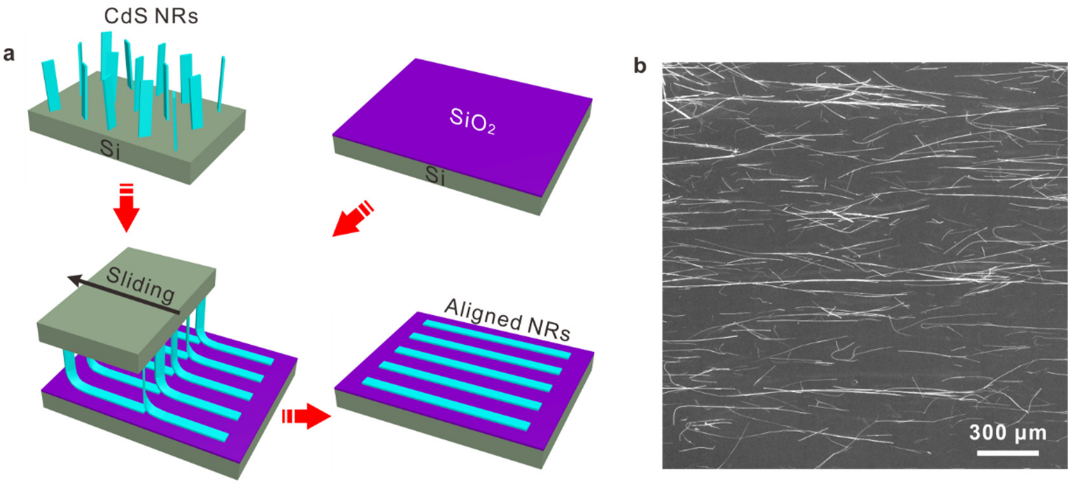


**Supplementary Figure 20** (a) Schematic illustration of contact printing process. (b) SEM image of semi-aligned CdS NRs on $SiO_2$/Si substrate.

The contact printing method involves the directional sliding of a Si growth substrate on top of a $SiO_2$/Si substrate. After the sliding step, the CdS NRs are detached from the Si substrate, resulting in the direct transfer of aligned CdS NRs to the $SiO_2$/Si substrate. To fabricate the single NR-based device, the density of aligned CdS NRs is controlled by adjusting the interaction force between the two substrates.

**Supplementary Note 1.** The exponential-association photoelectric conversion law for the MPT based on the charge accumulation model.

In a memory phototransistor (MPT), a large amount of electrons are trapped in the surface states of CdS NR in the initial OFF state. Under light irradiation, the photogenerated holes will be rapidly extracted from CdS NR under the surface electric filed and thus captured by electrons trapped at surface states, leaving photogenerated electrons in CdS NR. We assume that, at the pre-irradiation time $t = 0$, the number of electrons $N_{\mathrm{e}}(t)$ trapped at the surface state is $N_{\mathrm{e}}(0) = \mathrm{N}$ and the number of holes $N_{\mathrm{h}}(t)$ captured by the surface states is $N_{\mathrm{h}}(0) = 0$. At the pre-irradiation time *t*, holes of $N_{\mathrm{h}}(t)$ are captured by the surface states, leaving electrons of $N_{\mathrm{e}}(t) = \mathrm{N} - N_{\mathrm{h}}(t)$ in the surface states. During the next $\Delta t$ time, the number of holes generated by light irradiation should be $\mathrm{A} \cdot P \cdot \Delta t$, where A is a constant and $P$ is the light intensity. During this $\Delta t$ time, the number of holes captured by surface electrons $\Delta N_{\mathrm{h}}(t)$ should be proportional to the number of photogenerated holes $(\mathrm{A} \cdot P \cdot \Delta t)$ in the CdS NR and the number of remaining electrons $\left(\mathrm{N} - N_{\mathrm{h}}(t)\right)$ in the surface states, which can be approximatively given by $\Delta N_{\mathrm{h}}(t) = N_{\mathrm{h}}(t + \Delta t) - N_{\mathrm{h}}(t) = \mathrm{B} \cdot (\mathrm{A} \cdot P \cdot \Delta t) \cdot \left(\mathrm{N} - N_{\mathrm{h}}(t)\right)$, where B is a constant. When $\Delta t$ approaches 0, we get $dN_{\mathrm{h}}(t) = \mathrm{A} \cdot \mathrm{B} \cdot P \cdot \left(\mathrm{N} - N_{\mathrm{h}}(t)\right) \cdot dt$. Through the integral operation, the number of holes captured by surface electrons, $N_{\mathrm{h}}(t) = \mathrm{N} \cdot (1 - \mathrm{e}^{-\mathrm{A} \cdot \mathrm{B} \cdot P \cdot t})$, as well as the number of remaining surface electrons, $N_{\mathrm{e}}(t) = \mathrm{N} \cdot \mathrm{e}^{-\mathrm{A} \cdot \mathrm{B} \cdot P \cdot t}$, are obtained. As a result, the transport current in the phototransistor channel can be deduced as $I_{\mathrm{ph}} = \mathrm{C} \cdot (1 - \mathrm{e}^{-\mathrm{A} \cdot \mathrm{B} \cdot P \cdot t})$, where C is constant. Finally, the above formula can be simplified to $I_{\mathrm{ph}} = \mathrm{a} \cdot (1 - \mathrm{e}^{-\mathrm{b} \cdot P \cdot t})$, where a, b are constants, a represents the saturation value of $I_{\mathrm{ph}}$, and b·$P$ represents the rate constant of MPT approaching the saturation value.

During the derivation process, in order to simplify the physical model, we assumed that the

photogenerated holes do not need to diffuse a long distance to reach the traps due to the small thickness of NR and the existence of sufficient surface states, thus giving rise to a time-independent constant of B. In this case, the charge accumulation process is an exponential Debye evolution process, which could be regarded as a particular case ($\beta = 1$) of stretched exponential evolution process ($I_{\mathrm{ph}} = \mathrm{a} \cdot (1 - \mathrm{e}^{-(\mathrm{b} \cdot P \cdot t)^{\beta}})$.[10] For the case of the existence of few surface states or a large NR thickness, the photogenerated holes may have to diffuse a long distance to the traps. Therefore, in order to accurately describe the charge accumulation process, an exponent factor $\beta$ $(0 < \beta < 1)$ should be introduced to correct the derived exponential function.

**Supplementary Note 2.** Calculation of MPT performance.

In the photodetector, current responsivity $R$ can be evaluated by the following equation: $R = I_{\mathrm{ph}}/P \cdot \mathrm{A}$, where $I_{\mathrm{ph}}$, $P$, A are the photocurrent, light intensity, and the area of the device, respectively. The area of the device is equal to the area of CdS NR in the device channel. It can be given by $\mathrm{A} = \mathrm{L} \cdot \mathrm{W}$, where L and W are the channel length and width, respectively. Detectivity $D^*$ indicates the capability of a MPT to detect weak optical signals and can be calculated from the following expression: $D^* = (\mathrm{A}^{1/2} \cdot R)/(2\mathrm{q}I_{\mathrm{d}})^{1/2}$, where q is the elementary charge and $I_{\mathrm{d}}$ is the dark current. It is assumed that the dark current is dominated by the shot noise for estimating detectivity. Voltage responsivity $R_{\mathrm{V}}$ can be also utilized to evaluate the photoresponse of a phototransistor. It is defined by the following formula: $R_{\mathrm{V}} = \Delta V_{\mathrm{th}}/P \cdot \mathrm{A}$, where $\Delta V_{\mathrm{th}}$ is the shift of threshold voltage after the light irradiation. In Figure 3, a device with L and W of 23 and 0.38 μm, respectively, was measured. Here, we take the calculation at the light intensity of 190 nW $\mathrm{cm}^{-2}$ as an example. From the curves in Figure 3, the dark current and photocurrent at $V_{\mathrm{GS}}$=30 V is 0.2 pA and 3.10 μA, respectively, and $\Delta V_{\mathrm{th}}$ is 25.2 V. Based on the above equations and data, responsivity, detectivity, and voltage responsivity of the CdS MPT are deduced to be $1.86\times10^{8}$ A $\mathrm{W}^{-1}$, $2.18\times10^{22}$ Jones, and $1.52\times10^{15}$ V $\mathrm{W}^{-1}$, respectively.

**Supplementary Table 1.** Comparison of device performance of CdS NR MPT with other low-dimensional semiconductor nanostructure-based photodetectors.

| PD | Responsivity ($A\ W^{-1}$) | Detectivity (Jones) | Sensitivity @ light intensity ($W\ cm^{-2}$) | Reference |
|---|---|---|---|---|
| CdS NW | $5.2\times10^{6}$ | $1.7\times10^{18}$ | $10^{5}$ @ $3\times10^{-6}$ | [11] |
| CdS NW | $2.6\times10^{5}$ | $2.3\times10^{16}$ | $10^{3}$ @ $1\times10^{-5}$ | [12] |
| ZnO NW | $7\times10^{6}$ | $3.3\times10^{17}$ | 2 @ $5\times10^{-10}$ | [13] |
| SIZO/QDs | $6\times10^{3}$ | $7\times10^{13}$ | $10^{3}$ @ $2.55\times10^{-4}$ | [14] |
| Graphene | $1.5\times10^{3}$ | $1\times10^{11}$ | 0.05 @ $8\times10^{-3}$ | [15] |
| Graphene/$Ga_2O_3$ | 39.3 | $5.9\times10^{13}$ | 1.7 @ $4.67\times10^{-6}$ | [16] |
| Graphene/Si QDs | $1\times10^{9}$ | $1\times10^{13}$ | 25 @ $2\times10^{-7}$ | [17] |
| $MoS_2$ | $10^{4}$ | $7.7\times10^{13}$ | $10^{3}$ @ $2.5\times10^{-5}$ | [18] |
| $MoS_2$ | $7\times10^{4}$ | $3.5\times10^{14}$ | 4.5 @ $3\times10^{-8}$ | [19] |
| $WS_2$ | $10^{3}$ | $3.5\times10^{11}$ | $10^{3}$ @ $1\times10^{-3}$ | [20] |
| $WSe_2$/PbS QDs | $2\times10^{5}$ | $1\times10^{13}$ | 1.3 @ $2.5\times10^{-5}$ | [21] |
| $WSe_2$/$SnS_2$ | 244 | $1.29\times10^{13}$ | $10^{6}$ @ $3.77\times10^{-3}$ | [22] |
| $PtS_2$ | $1.56\times10^{6}$ | $2.9\times10^{11}$ | 0.4 @ $1.02\times10^{-4}$ | [23] |
| **CdS NR MPT** | **$3.8\times10^{9}$** | **$7.7\times10^{22}$** | **$10^{7}$ @ $6\times10^{-9}$** | **Our work** |

**Supplementary Table 2.** Comparison of device performance of the CdS NR MPT with other thin film and bulk crystal-based photodetectors.

| Photodetectors | Responsivity ($A\ W^{-1}$) | Detectivity (Jones) | Sensitivity @ light intensity ($W\ cm^{-2}$) | Reference |
|---|---|---|---|---|
| CdS film | $7.3\times10^{5}$ | $3.5\times10^{16}$ | 1.0 @ $1.9\times10^{-9}$ | [24] |
| ZnO film | $1.0\times10^{4}$ | $5.2\times10^{12}$ | 0.67 @ $3\times10^{-3}$ | [25] |
| $CH_3NH_3PbI_3$ film | 81 | $10^{11}$ | 17 @ $0.3\times10^{-6}$ | [26] |
| $CsPbBr_3$ film | 0.18 | $6.1\times10^{10}$ | $8\times10^{3}$ @ $10^{-3}$ | [27] |
| ZnSe bulk crystal | 4.4 | $1.4\times10^{11}$ | $4\times10^{3}$ @ 3 | [28] |
| $CH_3NH_3PbI_3$ bulk crystal | 953 | $3.2\times10^{14}$ | 223 @ $1.8\times10^{-3}$ | [29] |
| $MoS_2$ film/Si bulk crystal | 300 | $10^{13}$ | $8\times10^{3}$ @ $10^{-3}$ | [30] |
| **CdS NR MPT** | $\mathbf{3.8\times10^{9}}$ | $\mathbf{7.7\times10^{22}}$ | $\mathbf{10^{7}}$ **@** $\mathbf{6\times10^{-9}}$ | **Our work** |

## Supplementary References


1. Rantala, T. S., Golovanov, V. & Lantto, V. A cluster approach for the adsorption of oxygen and carbon monoxide on $SnO_2$ and CdS surfaces. *Sens. Actuators B-Chem.* **25**, 532-536 (1995).
2. Gu, Y. & Lauhon, L. J. Space-charge-limited current in nanowires depleted by oxygen adsorption. *Appl. Phys. Lett.* **89**, 143102 (2006).
3. Soudi, A., Hsu, C.-H. & Gu, Y. Diameter-dependent surface photovoltage and surface state density in single semiconductor nanowires. *Nano Lett.* **12**, 5111-5116 (2012).
4. Baidyaroy, S. & Mark, P. Analytical and experimental investigation of the effects of oxygen chemisorption on the electrical conductivity of CdS. *Surf. Sci.* **30**, 53-68 (1972).
5. Brillson, L. J. Observation of extrinsic surface states on (1120) CdS. *Surf. Sci.* **51**, 45-60 (1975).
6. Yan, Y. F., Al-Jassim, M. M. & Wei, S.-H. Oxygen-vacancy mediated adsorption and reactions of molecular oxygen on the ZnO(10-10) surface. *Phys. Rev. B* **72**, 161307 (2005).
7. Lin, Y. H., Wang, D. J., Zhao, Q. D., Li, Z. H., Ma, Y. D. & Yang, M. Influence of adsorbed oxygen on the surface photovoltage and photoluminescence of ZnO nanorods. *Nanotechnology* **17**, 2110 (2006).
8. Bertolazzi, S., Krasnozhon, D. & Kis, A. Nonvolatile memory cells based on $MoS_2$/graphene heterostructures. *ACS Nano* **7**, 3246-3252 (2013).
9. Lee, D., Hwang, E., Lee, Y., Choi, Y., Kim, J. S., Lee, S. & Cho, J. H. Multibit $MoS_2$

photoelectronic memory with ultrahigh sensitivity. *Adv. Mater.* **28**, 9196-9202 (2016).

10. Phillips, J. C. Stretched exponential relaxation in molecular and electronic glasses. *Rep. Prog. Phys.* **59**, 1133-1207 (1996).

11. Zheng, D. S., Wang, J. L., Hu, W. D., Liao, L., Fang, H. H., Guo, N., Wang, P., Gong, F., Wang, X. D., Fan, Z. Y., Wu, X., Meng, X. J., Chen, X. S. & Lu, W. When nanowires meet ultrahigh ferroelectric field-high-performance full-depleted nanowire photodetectors. *Nano Lett.* **16**, 2548-2555 (2016).

12. Zheng, D. S., Fang, H. H., Wang, P., Luo, W. J., Gong, F., Ho, J. C., Chen, X. S., Lu, W., Liao, L., Wang, J. L. & Hu, W. D. High-performance ferroelectric polymer side-gated CdS nanowire ultraviolet photodetectors. *Adv. Funct. Mater.* **26**, 7690-7696 (2016).

13. Liu, X., Gu, L. L., Zhang, Q. P., Wu, J. Y., Long, Y. Z. & Fan, Z. Y. All-printable band-edge modulated ZnO nanowire photodetectors with ultra-high detectivity. *Nat. Commun.* **5**, 4007 (2014).

14. Cho, K. S., Heo, K., Baik, C. W., Choi, J. Y., Jeong, H., Hwang, S. & Lee, S. Y. Color-selective photodetection from intermediate colloidal quantum dots buried in amorphous-oxide semiconductors. *Nat. Commun.* **8**, 840 (2017).

15. Liu, C.-H., Chang, Y.-C., Norris, T. B. & Zhong, Z. H. Graphene photodetectors with ultra-broadband and high responsivity at room temperature. *Nat. Nanotechnol.* **9**, 273-278 (2014).

16. Kong, W. Y., Wu, G. A., Wang, K. Y., Zhang, T. F., Zou, Y. F., Wang, D. D. & Luo, L. B. Graphene-$\beta$-$Ga_2O_3$ heterojunction for highly sensitive deep UV photodetector application. *Adv. Mater.* **28**, 10725-10731 (2016).

17. Ni, Z. Y., Ma, L. L., Du, S. C., Xu, Y., Yuan, M., Fang, H. H., Wang, Z., Xu, M. S., Li, D. S., Yang, J. Y., Hu, W. D., Pi, X. D. & Yang, D. R. Plasmonic silicon quantum dots enabled high-sensitivity ultrabroadband photodetection of graphene-based hybrid phototransistors. *ACS Nano* **11**, 9854-9862 (2017).

18. Kufer, D. & Konstantatos, G. Highly sensitive, encapsulated $MoS_2$ photodetector with gate controllable gain and speed. *Nano Lett.* **15**, 7307-7313 (2015).

19. Huo, N. J. & Konstantatos, G. Ultrasensitive all-2D $MoS_2$ phototransistors enabled by an out-of-plane $MoS_2$ PN homojunction. *Nat. Commun.* **8**, 572 (2017).

20. Gong, F., Luo, W. J., Wang, J. L., Wang, P., Fang, H. H., Zheng, D. S., Guo, N., Wang, J. L., Luo, M., Ho, J. C., Chen, X. S., Lu, W., Liao, L. & Hu, W. D. High-sensitivity floating-gate phototransistors based on $WS_2$ and $MoS_2$. *Adv. Funct. Mater.* **26**, 6084-6090 (2016).

21. Hu, C., Dong, D. D., Yang, X. K., Qiao, K. K., Yang, D., Deng, H., Yuan, S. J., Khan, J., Lan, Y., Song, H. S. & Tang, J. Synergistic effect of hybrid PbS quantum dots/2D-$WSe_2$ toward high performance and broadband phototransistors. *Adv. Funct. Mater.* **27**, 1603605 (2017).

22. Zhou, X., Hu, X. Z., Zhou, S. S., Song, H. Y., Zhang, Q., Pi, L. J., Li, L., Li, H. Q., Lü, J. T. & Zhai, T. Y. Tunneling diode based on $WSe_2$/$SnS_2$ heterostructure incorporating high detectivity and responsivity. *Adv. Mater.* **30**, 1703286 (2018).

23. Li, L., Wang, W. K., Chai, Y., Li, H. Q., Tian, M. L. & Zhai, T. Y. Few-layered $PtS_2$ phototransistor on h-BN with high gain. *Adv. Funct. Mater.* **27**, 1701011 (2017).

24. Lin, K.-T., Chen, H.-L., Lai, Y.-S., Liu, Y.-L., Tseng, Y.-C. & Lin, C.-H. Nanocrystallized CdS beneath the surface of a photoconductor for detection of UV light with picowatt sensitivity. *ACS Appl. Mater. Interfaces* **6**, 19866-19875 (2014).

25. Roul, B., Pant, R., Chirakkara, S., Chandan, G., Nanda, K. K. & Krupanidhi, S. B. Enhanced UV photodetector response of ZnO/Si with AlN buffer layer. *IEEE Trans. Electron Devices* **64**, 4161-4166 (2017).

26. Hu, W., Huang, W., Yang, S. Z., Wang, X., Jiang, Z. Y., Zhu, X. L., Zhou, H., Liu, H. J., Zhang, Q. L., Zhuang, X. J., Yang, J. L., Kim, D. H. & Pan, A. L. High-performance flexible photodetectors based on high-quality perovskite thin films by a vapor-solution method. *Adv. Mater.* **29**, 1703256 (2017).

27. Li, X. M., Yu, D. J., Cao, F., Gu, Y., Wei, Y., Wu, Y., Song, J. Z. & Zeng, H. B. Healing all-inorganic perovskite films via recyclable dissolution-recyrstallization for compact and smooth carrier channels of optoelectronic devices with high stability. *Adv. Funct. Mater.* **26**, 5903-5912 (2016).

28. Sirkeli, V. P., Yilmazoglu, O., Hajo, A. S., Nedeoglo, N. D., Nedeoglo, D. D., Preu, S., Küppers, F. & Hartnagel, H. L. Enhanced responsivity of ZnSe-based metal-semiconductor-metal near-ultraviolet photodetector via impact ionization. *Phys. Status Solidi RRL* **12**, 1700418 (2017).

29. Lian, Z. P., Yan, Q. F., Lv, Q. R., Wang, Y., Liu, L. L., Zhang, L. J., Pan, S. L., Li, Q., Wang, L. D. & Sun, J.-L. High-performance planar-type photodetector on (100) facet of $MAPbI_3$ single crystal. *Sci. Rep.* **5**, 16563 (2015).

30. Wang, L., Jie, J. S., Shao, Z. B., Zhang, Q., Zhang, X. H., Wang, Y. M., Sun, Z. & Lee, S.-T. $MoS_2$/Si heterojunction with vertically standing layered structure for ultrafast, high-detectivity, self-driven visible-near Infrared photodetectors. *Adv. Funct. Mater.* **25**, 2910-2919 (2015).
